# Quantifying the Severity of Adverse Drug Reactions Using Social Media: Network Analysis

**DOI:** 10.2196/27714

**Published:** 2021-10-21

**Authors:** Adam Lavertu, Tymor Hamamsy, Russ B Altman

**Affiliations:** 1 Biomedical Informatics Training Program Stanford University Stanford, CA United States; 2 Department of Biomedical Data Science Stanford University Stanford, CA United States; 3 Center for Data Science New York University New York, NY United States; 4 Department of Bioengineering Stanford University Stanford, CA United States; 5 Department of Genetics Stanford University Stanford, CA United States

**Keywords:** social media for health, pharmacovigilance, adverse drug reactions, machine learning, network analysis, word embeddings, drug safety, social media

## Abstract

**Background:**

Adverse drug reactions (ADRs) affect the health of hundreds of thousands of individuals annually in the United States, with associated costs of hundreds of billions of dollars. The monitoring and analysis of the severity of ADRs is limited by the current qualitative and categorical systems of severity classification. Previous efforts have generated quantitative estimates for a subset of ADRs but were limited in scope because of the time and costs associated with the efforts.

**Objective:**

The aim of this study is to increase the number of ADRs for which there are quantitative severity estimates while improving the quality of these severity estimates.

**Methods:**

We present a semisupervised approach that estimates ADR severity by using social media word embeddings to construct a lexical network of ADRs and perform label propagation. We used this method to estimate the severity of 28,113 ADRs, representing 12,198 unique ADR concepts from the Medical Dictionary for Regulatory Activities.

**Results:**

Our Severity of Adverse Events Derived from Reddit (SAEDR) scores have good correlations with real-world outcomes. The SAEDR scores had Spearman correlations of 0.595, 0.633, and −0.748 for death, serious outcome, and no outcome, respectively, with ADR case outcomes in the Food and Drug Administration Adverse Event Reporting System. We investigated different methods for defining initial seed term sets and evaluated their impact on the severity estimates. We analyzed severity distributions for ADRs based on their appearance in boxed warning drug label sections, as well as for ADRs with sex-specific associations. We found that ADRs discovered in the postmarketing period had significantly greater severity than those discovered during the clinical trial (*P*<.001). We created quantitative drug-risk profile (DRIP) scores for 968 drugs that had a Spearman correlation of 0.377 with drugs ranked by the Food and Drug Administration Adverse Event Reporting System cases resulting in death, where the given drug was the primary suspect.

**Conclusions:**

Our SAEDR and DRIP scores are well correlated with the real-world outcomes of the entities they represent and have demonstrated utility in pharmacovigilance research. We make the SAEDR scores for 12,198 ADRs and the DRIP scores for 968 drugs publicly available to enable more quantitative analysis of pharmacovigilance data.

## Introduction

### Background

Adverse drug reactions (ADRs) are among the leading causes of mortality and morbidity in the United States, affecting hundreds of thousands of people and costing more than US $500 billion every year in the United States alone [1,2]. An ADR is characterized as “an appreciably harmful or unpleasant reaction resulting from an intervention related to the use of a medicinal product” [3]. A drug’s ADRs are primarily derived from clinical trial data and augmented through postmarketing surveillance [4]. The ADR labeling process for each drug is based on the frequency of the ADRs in the treated populations and the severity of the outcomes associated with each ADR. The severity of an ADR is traditionally classified into one of three categories characterized by where it appears on the label: boxed warning, warnings and precautions, or adverse reactions, listed in decreasing order of associated severity [5]. Boxed warnings refer to “serious warnings, particularly those that lead to death or serious injury.” Under the existing system, death is severe enough to warrant a boxed warning, but so is restlessness; therefore, it would be useful for prescribers, patients, and researchers to have systems for recognizing that death is more severe than restlessness [6]. Similarly, warnings, precautions, and adverse reactions include a diverse spectrum of ADRs, and it can be difficult for patients, prescribers, and researchers to compare the risk profiles of different drugs.

The existing categorical definitions of ADR severity limit the ability of researchers and regulators to apply quantitative methods to regulatory and pharmacovigilance efforts. For instance, tracking the regulatory performance of drug safety efforts is primarily achieved through a categorical analysis of ADR case outcomes and drug label changes. The creation of a quantitative numerical scale that provides a relative severity score for each ADR would enable these analyses to leverage the powerful tools of quantitative decision and utility theory.

The pioneering study by Tallarida et al [7] examined the ability to create a continuous ADR severity scale. A total of 53 physicians were interviewed and asked to estimate the acceptable probabilities in a set of scenarios specifying risk-benefit trade-offs. These probabilities were then used to define a set of equations and the expected benefit to the patient in each scenario. Solving the system of equations resulted in numerical severity weights that were relative to the lowest ADR severity. The effort took approximately 45 minutes per interview and resulted in relative scores for seven ordinal categories of ADR severity.

The study by Gottlieb et al [8] expanded this ADR severity ranking task using the crowdsourcing platform Amazon Mechanical Turk. Individual workers were presented with a pair of ADRs and a link to more information on these ADRs and asked to select the ADR that they perceived to be more severe. Over 146 person-days, 2589 workers produced severity comparisons for 126,512 ADR pairs composed from 2929 unique ADRs. A linear programming algorithm was used to create a unified ADR severity ranking. To validate the ranking, the ADR severities were correlated with ADR case outcomes from the Food and Drug Administration Adverse Event Reporting System (FAERS). The highest Spearman correlation, ρ=0.53, was associated with the proportion of cases resulting in death. This effort drastically increased the number of ADRs included in the severity ranking but was costly in terms of human time, labor, and money.

Recent work in natural language processing has resulted in numerous methods for creating vectorized word representations (also known as *embeddings*) that capture semantic meaning in a dense numerical representation [9,10]. These methods rely on the distributional hypothesis that word meaning is captured by the contexts in which a word appears [11]. The practical implication of the distributional hypothesis is that training a model to predict word context (ie, co-occurring word pairs) results in model weights that capture the meaning of the word. These model weights can then be used as a numerical representation of a given word.

Word embeddings learned on social media data sets have been deployed for pharmacovigilance previously but not for the purpose of exploring ADR severity [12]. By using a social media corpus generated by the general public, especially on pseudoanonymous social media platforms such as Reddit, researchers can capture meanings that reflect people’s unfiltered experiences of, and opinions about, health and disease [13,14]. Investigating the utility, benefits, and challenges of different social media platforms and methods for pharmacovigilance has been an area of active research [15]. Previous research on Twitter data annotated samples of tweets for personal medication intake versus individuals simply mentioning a drug [16,17]. Together, the 2 studies found that approximately 40% of the tweets mentioning a drug indicated that the individual tweeting was possibly personally taking that medication. Here, we focused on ADRs, but it is likely that the individuals discussing ADRs on social media have more direct experience with the ADRs, either by being directly affected by the ADRs or being informed about the ADR experience by a close relation, than individuals selected from a pool of crowdworkers. Word embeddings trained on a corpus generated by the general public can then be leveraged as a metric for public opinion in a similar fashion to previous crowdsourced approaches. We used the RedMed embeddings trained on more than 580 million health-enriched Reddit comments from more than 10 million users [18]. These numbers dwarf the number of votes gathered in a typical crowdsourcing experiment and are therefore potentially more indicative of a representative population’s perception of ADRs than traditional survey-based methods.

### Objective

In this study, we used publicly available word embeddings and a network-based label propagation method to estimate the severity of 12,198 ADR concepts from Medical Dictionary for Regulatory Activities (MedDRA) terminology. The resulting Severity of Adverse Events Derived from Reddit (SAEDR) scores were validated against human rankings as well as FAERS case outcomes. We used System Organ Classes (SOCs) and other groupings within the MedDRA to examine how the SAEDR severity scores differ at various levels of abstraction and within ADR categories. We used the SAEDR scores to compare the severity among ADRs in different sections of drug labels, ADRs with disproportionate rates between sexes, and ADRs discovered at different stages of drug development. We combined the SAEDR scores with frequency information from Side Effect Resource (SIDER)–derived drug labels to generate drug-specific aggregate side effect severity scores [19]. The SAEDR scores enabled new analyses that were not possible with the existing categorical classifications.

## Methods

### Data Sources and Preparation

#### ADR Terms and Phrases

We sourced our ADR phrases and their synonyms from the *lowest-level terms* within the terminology of the MedDRA, version 22 [20]. We filtered terms based on their semantic types within the Unified Medical Language System Metathesaurus [21] and retained terms of the following semantic types: *Disease or Syndrome* (T047), *Finding* (T033), *Neoplastic Process* (T191), *Injury or Poisoning* (T037), *Pathologic Function* (T046), *Sign or Symptom* (T184), *Mental or Behavioral Dysfunction* (T048), and *Congenital Abnormality* (T019).

#### Word-Embedding Model

We used the word-embedding model from the RedMed project [18]. This model was selected because it was directly optimized for medical term similarity and was trained on a corpus of Reddit comments generated by the public and preprocessed to maximize the inclusion of ADR terms. ADRs that were a phrase such as “abdominal pain” were represented using the average embedding of all terms within the phrase. In previous work, not presented here, we found that the use of average embeddings, for example, “abdominal” + “pain”/2, produced better results than the use of embedded phrases, for example, “abdominal pain.”

#### Gottlieb Severity Data

We used the crowdsourced severity estimates included within the supplement of the study by Gottlieb et al [8], which included 2929 ADRs with rank scores.

#### FAERS Data

All adverse drug event data were downloaded in JSON format from the openFDA website, which includes data from both the Legacy Adverse Event Reporting System and the FAERS. Data were retrieved on August 8, 2020, and included adverse event case reports up to June 30, 2020. Drug reactions were normalized to the MedDRA, version 22. We filtered to unique case IDs using the provided duplicated flag to remove duplicate reports and case reports that did not originate in the United States. Case outcomes were normalized according to the following schema: “Death”: {“Death”}, “Serious Outcome”: {“Life-Threatening,” “Hospitalization,” “Other Serious,” “Required Intervention”}, and “Disability”: {“Congenital Anomaly,” “Disability”}. We created an additional outcome category denoted *No Outcome* for cases with no reported outcome. We felt the need to create this category because the FAERS only allows for the reporting of serious case outcomes, and there is information in the absence of a serious outcome being reported. The outcome proportions for each ADR were calculated by dividing the number of cases reporting that ADR for a particular outcome by the total number of cases reporting that ADR.

#### FAERS Severity Rankings

For the ranking of ADRs based on the FAERS data, we ranked ADRs based on their marginal likelihood to be included in a case with death or serious outcome as the reported case outcome. This was calculated by dividing the number of cases with the ADR that resulted in death or serious outcome by the number of cases without the ADR that resulted in these outcomes.

### Semisupervised Severity Propagation

#### Overview

**Figure 1:**
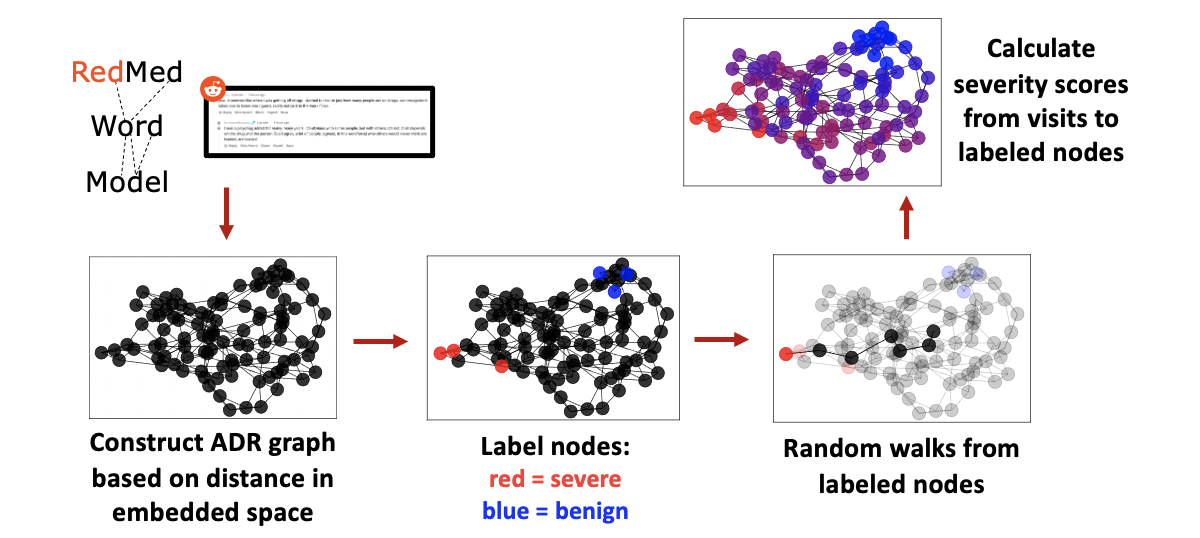
Overview of the network method for estimating ADR severity from word embeddings. Word embeddings for 28,113 ADR terms and phrases were extracted from the RedMed word-embedding model. A network is constructed based on nearest-neighbors in the embedding space. A subset of nodes is labeled as severe or benign ADRs, and random walks from these labeled nodes are conducted. The Severity of Adverse Events Derived from Reddit scores for each ADR are calculated based on the relative number of encounters in random walks initiated from severe versus benign nodes. ADR: adverse drug reaction.

Given a word-embedding model and a set of initial, potentially noisy, seed-word labels for the severe and benign categories, we sought to propagate severity information over the rest of the vocabulary, similar to the sentiment propagation methods described in the study by Hamilton et al [22]. A graphical overview of this method is presented in Figure 1.

#### Lexical Network Creation

The network was constructed by connecting words (nodes) with edges to their *k-nearest neighbors* based on the cosine distance between their embeddings. The corresponding edge weight was set to the cosine similarity of the two words (ie, the edge weight increases with term similarity).

#### Seed Selection From Terminology

An initial set of ranked seed terms was labeled. Biomedical terminologies and ontologies often contain many terms that are highly similar to each other. To reduce the effect of lexical similarities on label propagation, we filtered the benign and severe seed terms based on the edit ratio among the terms in each list, as defined by the ratio function in the python-Levenshtein package [23]. Only terms that had an edit ratio >0.5 from a higher-ranking term were included in the list, resulting in a reduced list of lexically distinct seed terms. Both benign and severe seed term lists were truncated to the minimum seed term list size of both seed sets.

For instance, if the severe seed term list had [“death,” “cardiac arrest acute,” “cardiac arrest”] in rank order and the benign seed term list had [“dry skin,” “yawning,” “cold sweat”] in rank order, then, after lexical filtering, the severe seed term list would be [“death,” “cardiac arrest acute”], and the benign seed term list would remain unchanged. The final seed term lists would be truncated to the minimum seed term list size, resulting in a severe seed term list [“death,” “cardiac arrest acute”] and a benign seed term list [“dry skin,” “yawning”].

#### Random Walks From Seed Nodes

Given a network and a set of seed nodes, we modified code from *node2vec* [24] to perform 5000 weighted random walks of length 200 from each seed node. We selected the number of random walks to ensure that all nodes within the graph were visited a nonzero number of times. We noted that this value needs to be empirically determined and will likely change based on the size and structure of the network.

#### SAEDR Score Calculation

The SAEDR score of a given node *u* is calculated using the following formula:

u_SAEDR_ = s_u_ / s_u_ + b_u_

In the formula, s_u_ and b_u_ are the respective number of times that node *u* is encountered in a random walk from a severe node or a benign node. If node *u* was contained within one of our seed node sets, self-visits during the random walks were excluded from the calculation. To increase the robustness of these score estimates, we performed 10,000 iterations of bootstrap sampling [25] of the random walks, and the average of these bootstrap estimates was calculated. The SAEDR scores from multiple seed sets were averaged at the preferred term (PT) level, and the final combined scores were normalized to a zero to one range.

### Hyperparameter Tuning for Severity Propagation

We randomly split the crowdsourced severity data into training (1778/2369, 75.05%) and test (591/2369, 24.95%) sets, with 284 ADRs dropped because of mapping. We performed a grid search over the number of neighbors used to construct the lexical network {2, 5, 10, 15, 20, 25, 30} and the percentage of nodes to label for the severe and benign seed nodes {2, 5, 10,15, 20, 25}. For example, an individual run would use the 25 nearest neighbors and 5% at each end of the training severity rank (ie, the top 5% most severe and bottom 5% least severe ADRs). We ultimately selected the parameters with the highest Spearman correlation with the training data.

### ADR Discovery Group Analysis

We analyzed the SAEDR score distributions of several different categories of ADRs.

#### ADRs Included in a Boxed Warning

We downloaded the counts for the number of appearances of ADRs in the boxed warning section of drug labels from the supplement of the study by Wu et al [26]. ADRs that appear on a drug label based on their appearance in the SIDER but were not included in the list provided in the study by Wu et al [26] were considered to not have appeared in a boxed warning section [19].

#### ADRs With Disproportionate Reporting Between Sexes

A set of ADRs shown to have sex differences was identified from the supplement of the study by Chandak et al [27]. We filtered out ADRs considered borderline (mean log(ROR)<0.4 and mean log(ROR)>−0.4), as defined in the original study by Chandak et al [27]. Their study reported ADRs at the high-level group term (HLGT) level within the MedDRA, whereas our severity estimates were generated at the PT level. There are many PT ADRs per HLGT; therefore, all PTs within the specified HLGT terms were averaged to create a single SAEDR score for that HLGT. We did not use the drug information except to note distinct instances of sex-specific HLGTs (ie, HLGTs associated multiple times with different drugs each time).

#### ADRs Discovered at Different Stages of Drug Development

ADRs discovered at the clinical trial stage were identified based on their inclusion in a drug label in the SIDER with a non-postmarketing ADR frequency [19]. Postmarketing ADRs were identified as those included on drug labels with postmarketing frequency information and no clinical trial frequency information. OFFSIDES and TWOSIDES ADRs were discovered from the FAERS data by looking at disproportionate reporting after correcting for the effects of demographics and other case information [28]. The OFFSIDES and TWOSIDES sets are the results of a study by Tatonetti et al [28] in which propensity score matching was used to identify matched cases and controls among the FAERS cases and to also identify potentially novel ADRs based on disproportional reporting metrics. This was done to discover ADRs that were associated with a single drug, OFFSIDES, as well as ADRs that were potentially the result of drug-drug interactions, TWOSIDES. We considered both OFFSIDES and TWOSIDES ADRs discovery-stage postmarketing ADRs because they have not yet been included in the drug labeling.

### Drug-Risk Profile Scores

The drug-risk profile (DRIP) scores were calculated based on our SAEDR score and the frequency of each ADR for a given drug. The DRIP score for a given drug is the sum of the severity multiplied by the frequency of each ADR on the drug label. Drug ADR frequency information was retrieved from the SIDER [19]. In situations where multiple ADR frequencies were indicated, 1000 frequency estimates were sampled from a uniform distribution with a lower bound at the minimum-reported frequency and an upper bound at the maximum-reported frequency. When there was no frequency information, we sampled 1000 estimates from a uniform distribution over the interval of 0.001 to 0.01. The final DRIP score was the average score across 1000 samples.

## Results

### Network Statistics

We used the ADR terms and phrases from the MedDRA, version 22.0, lowest-level terms [20] as the initial lexicon for the lexical ADR network. Although the FAERS uses the MedDRA to encode ADRs, versioning differences over the years have resulted in some FAERS ADRs not being included in the MedDRA. We were able to generate embeddings for 92.35% (2450/2653) of the unique crowdsourced ADRs, 100% (14,045/14,045) of the unique FAERS ADRs, and 68.72% (28,113/40,905) of all filtered MedDRA terms. This resulted in a final lexical network with 28,113 nodes, representing 12,198 MedDRA PTs.

### SAEDR Score Performance

We compared our severity estimates using crowdworker-ranked seeds, FAERS-ranked seeds, and the average severity estimate across the two seed sets with two different ADR rankings: (1) a held-out test set of crowdworker-ranked ADRs (n=591) and (2) ADRs ranked by case outcome statistics in the FAERS database.

The crowdworker-seeded severity estimates had the highest training performance with a 25 nearest-neighbors graph and using 10% of the most and least severe ADRs as seeds. The Spearman correlation with the crowdworker test set was 0.747 (*P*<.001). The Spearman correlations for events with at least 100 reports in the FAERS were 0.595, 0.616, and −0.732 for death, serious outcome, and no outcome, respectively, with *P*<.001 for all (Figures S1 and S2 of Multimedia Appendix 1).

The FAERS-seeded severity estimates had the highest training performance with a 10 nearest-neighbors graph and using 10% of the most and least severe ADRs as seeds. The Spearman correlation with the crowdworker test set was 0.587. As no information from the crowdworker rankings was used to select these seeds, we can also report the Spearman correlation with the entire set of crowdworker ADRs: 0.765. The Spearman correlations for events with at least 100 reports in the FAERS were 0.509, 0.557, and −0.656 for death, serious outcome, and no outcome, respectively, with *P*<.001 for all (Figures S1 and S2 of Multimedia Appendix 1).

**Figure 2:**
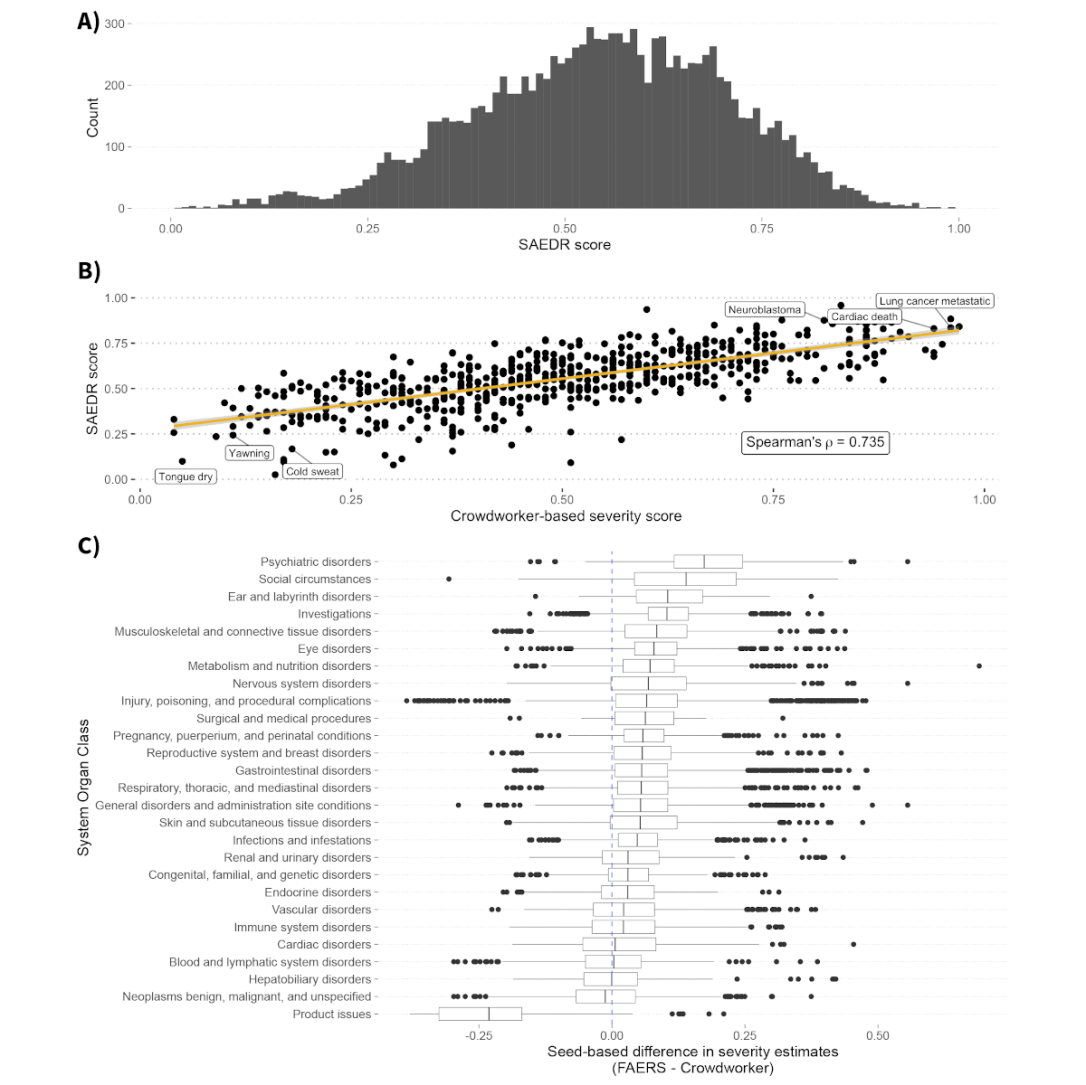
Comparison of SAEDR scores and crowdworker severity estimates. (A) Histogram of SAEDR scores for 12,198 adverse drug reactions (ADRs). (B) Crowdworker severity estimates (x-axis) versus SAEDR scores (y-axis) for a test set of 591 ADRs. The SAEDR scores showed a strong Spearman correlation, ρ=0.735, with the human crowdworker rankings. We noted that this correlation is greater than the interrater correlation, ρ=0.71, reported in the original crowdsource study by Gottlieb et al [8]. A select set of the least and most severe ADRs based on the SAEDR score has been annotated. (C) Differences between severity estimates seeded with FAERS rankings and those seeded with crowdworker rankings (x-axis) for different System Organ Class groups (y-axis). The dashed blue line indicates where the severity would be the same for both estimates. FAERS: Food and Drug Administration Adverse Event Reporting System; SAEDR: Severity of Adverse Events Derived from Reddit.

The SAEDR score—the average of the two severity estimates—had a Spearman correlation with the crowdworker test set of 0.735 (Figure 2). The Spearman correlations for events with at least 100 reports in the FAERS were 0.595, 0.633, and −0.748 for death, serious outcome, and no outcome, respectively (Figures S1 and S2 of Multimedia Appendix 1). All these correlations were statistically significant (*P*<.001).

### Seed-Ranking Comparisons

We examined the effects of seeding on the severity estimates of individual ADRs. Most ADRs were not substantially changed by the use of different seeds. We aggregated ADR severity differences at the MedDRA SOC level and found that the FAERS seeds led to increased severity estimates for *Psychiatric Disorders* and *Social Circumstances* (Figure 2). The most drastic shift in values was the decrease in severity of *Product Issue*–related ADRs using FAERS seeds compared with their severity when using crowdworker-based seeding.

### SOC Severity Rankings

**Figure 3:**
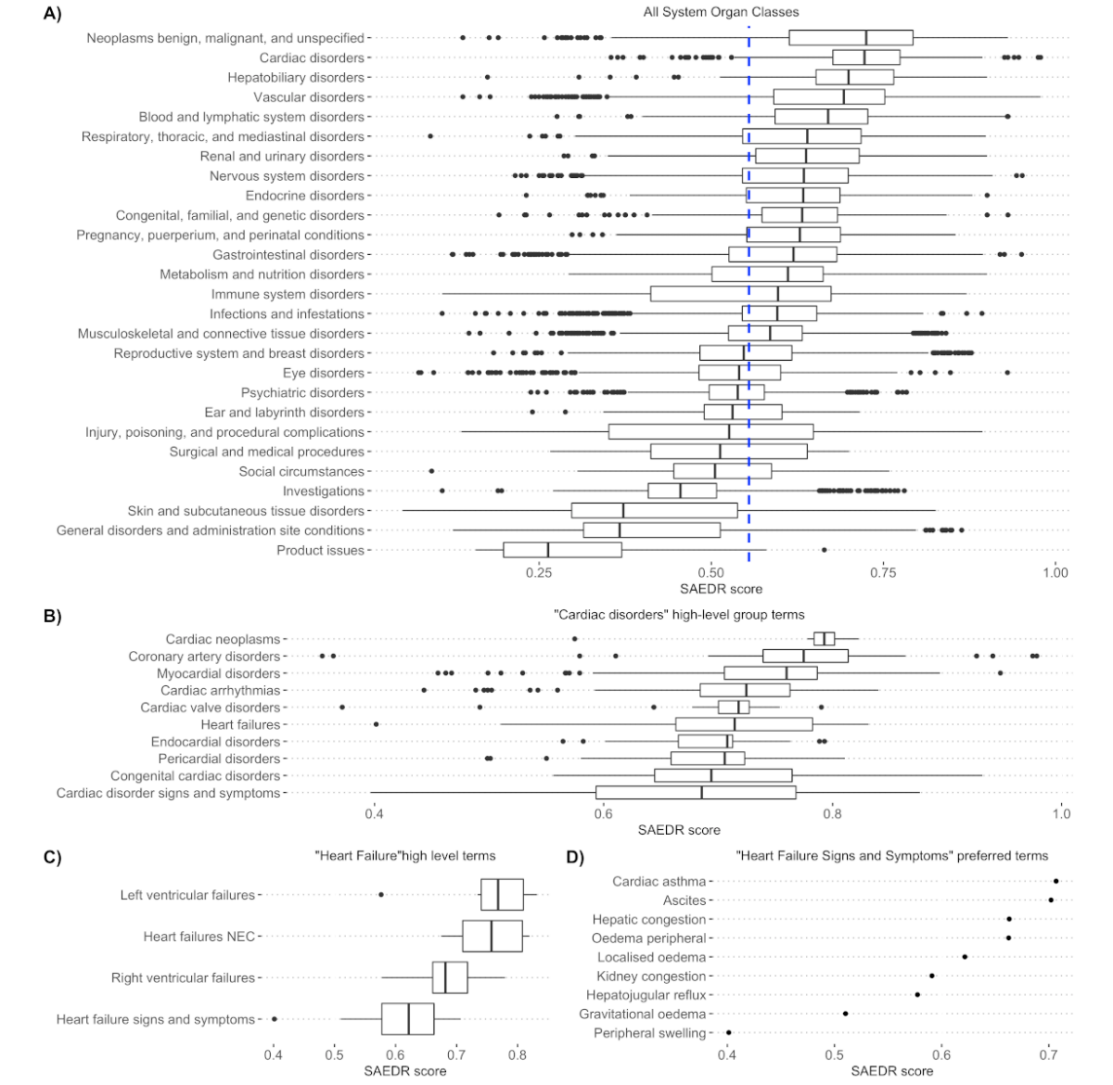
Severity of adverse drug reactions (ADRs) at different group resolutions. (A) SAEDR score (x-axis) for 11,981 ADRs with System Organ Class (SOC) groups (y-axis). The dashed blue line indicates an overall median SAEDR score of 0.554. The SAEDR score distributions indicate that cancers and cardiac-related side effects are considered the most severe, with product issues being the least severe. (B) Severity distributions of the Medical Dictionary for Regulatory Activities high-level group terms (HLGTs) within the Cardiac Disorders SOC. There are large differences in severity within these term groups. (C) High-level terms within the Heart Failure HLGTs show a tighter distribution of severity, with "Heart Failure Signs and Symptoms" being the least severe within this group. (D) Individual side effects within the signs and symptoms of heart failure suggest cardiac asthma and ascites (accumulation of fluid in the peritoneal cavity) are the most severe symptoms, and peripheral swelling is the least severe symptom NEC: necrotizing enterocolitis; SAEDR: Severity of Adverse Events Derived from Reddit.

We compared the severity of ADRs based on the MedDRA SOC groupings as a qualitative evaluation (Figure 3). We found the ADRs related to cancer (neoplasms), cardiac, and liver (hepatobiliary) to be the most severe, with high SAEDR scores, whereas skin, general disorders, and product issues were considered the least severe, with low SAEDR scores. We performed a qualitative examination of ADRs at various other levels. We compared the severity distributions between the ADRs describing benign neoplasms and those describing malignant neoplasms (Figure S3 of Multimedia Appendix 1). A one-sided *t* test found malignant neoplasms to be significantly more severe than benign neoplasms based on their SAEDR scores (*P*<.001). We found a wide range in terms of severity within the different levels of the MedDRA ADR term groupings (Figure 3). We noted that among ADRs in the *Cardiac Disorders* HLGT, cardiac neoplasms have the highest SAEDR scores, whereas signs and symptoms of cardiac disorder have the lowest relative SAEDR scores (Figure 3). When examining the level of individual ADRs within the *Heart Failure Signs and Symptoms* HLGT, we found that the ADRs that we would judge to be more severe were ranked higher than those that are more general and considered less severe.

### Severity of ADRs Grouped by Labeling Section, Sex Effects, and Time Point of Discovery

**Figure 4:**
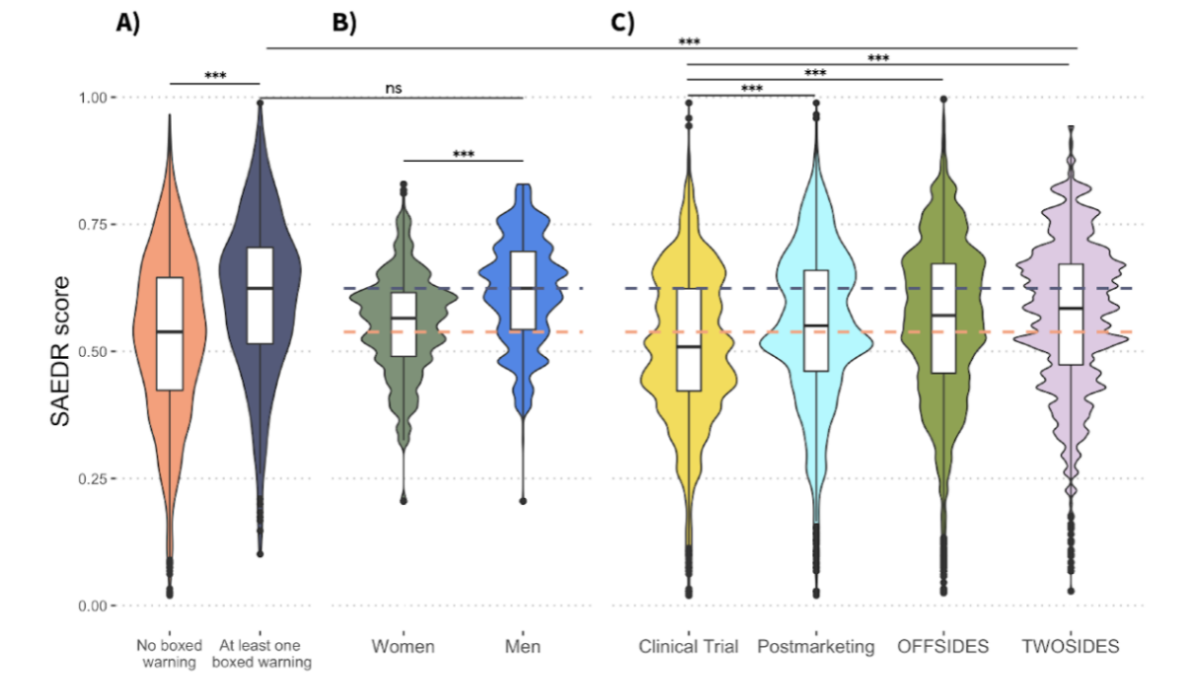
Differences in adverse drug reaction (ADR) severity between ADR groupings and discovery periods: ADR groups (x-axis) versus SAEDR scores (y-axis). The grey dashed line indicates the median severity, 0.624, of the ADRs that have been included in a boxed warning. The orange boxed line indicates the median severity, 0.538, of the ADRs that appear on a drug label but have not been included in a boxed warning. (A) The ADRs that were listed as a black box warning at least once (n=356) were significantly more severe than those that have not appeared in a black box warning (n=3305). (B) The ADRs that are disproportionately reported for men (n=56,405) are significantly more severe than those disproportionately reported for women (n=50,801). There was no significant difference (ns) in severity between the ADRs included in black box warnings and those disproportionately reported for men. (C) The ADRs discovered in the postmarketing period (n=11,506) are significantly more severe (*** indicate *P*<.001) than those discovered in the clinical trials (n=35,450). The ADRs identified in the postmarketing period through OFFSIDES (n=350,631) and the postmarketing polypharmacy ADRs identified through TWOSIDES (n=4,210,513) are significantly more severe than those discovered in the clinical trials. The severity of all postmarketing ADR groups is significantly less than the severity of the ADRs that have appeared in a black box warning. SAEDR: Severity of Adverse Events Derived from Reddit.

On the basis of our estimate, we calculated that the ADRs that have never been included in a boxed warning had a median SAEDR score of 0.538, whereas the ADRs that had been included in at least one boxed warning had a median SAEDR score of 0.624. We found that among the ADRs that have appeared on a drug label, those that have been included in at least one boxed warning were significantly more severe (*P*<.001) than the ADRs that have never been included in a boxed warning section based on a one-sided *t* test (Figure 4).

We found that the ADR HLGTs that are disproportionately reported for men were significantly more severe (*P*<.001) than those disproportionately reported for women (Figure 4). The HLGTs disproportionately experienced by men were not significantly different from the severity of the ADRs that have been included in at least one boxed warning, *P*=.13, based on a one-sided *t* test.

We examined the severity of the ADRs that were found during the clinical trial versus the severity of those discovered in the postmarketing period based on the SIDER label annotations. We found that the ADRs discovered in the postmarketing period had significantly higher severity (*P*<.001) than the ADRs discovered in the clinical trials based on a one-sided *t* test (Figure 4). We compared the severity of the clinical trial ADRs with the severity of those discovered in the postmarketing period using FAERS data. The study by Tatonetti et al [28] identified two different sets of ADRs: OFFSIDES is a set of ADRs disproportionately reported for a drug, whereas TWOSIDES is a set of ADRs disproportionately reported for a pair of drugs being taken concurrently, after correcting for case demographics and other information. The severity distribution of ADRs in both OFFSIDES and TWOSIDES was significantly higher (*P*<.001) than the severity distribution discovered in the clinical trials based on a one-sided *t* test (Figure 4). We found that the ADRs associated with polypharmacy, through TWOSIDES, had the highest severity of the postmarketing ADRs. All postmarketing ADRs were significantly lower in severity than the boxed warning ADRs based on a one-sided *t* test.

### DRIP Score Analysis

We calculated the DRIP scores for 968 drugs using SIDER label data, with a resulting median DRIP score of 0.439. The Spearman correlation between the drugs ranked by the proportion of FAERS cases, with that drug as the primary suspect that resulted in death, and the drugs ranked by our DRIP scores was 0.377, *P*<.001.

**Figure 5:**
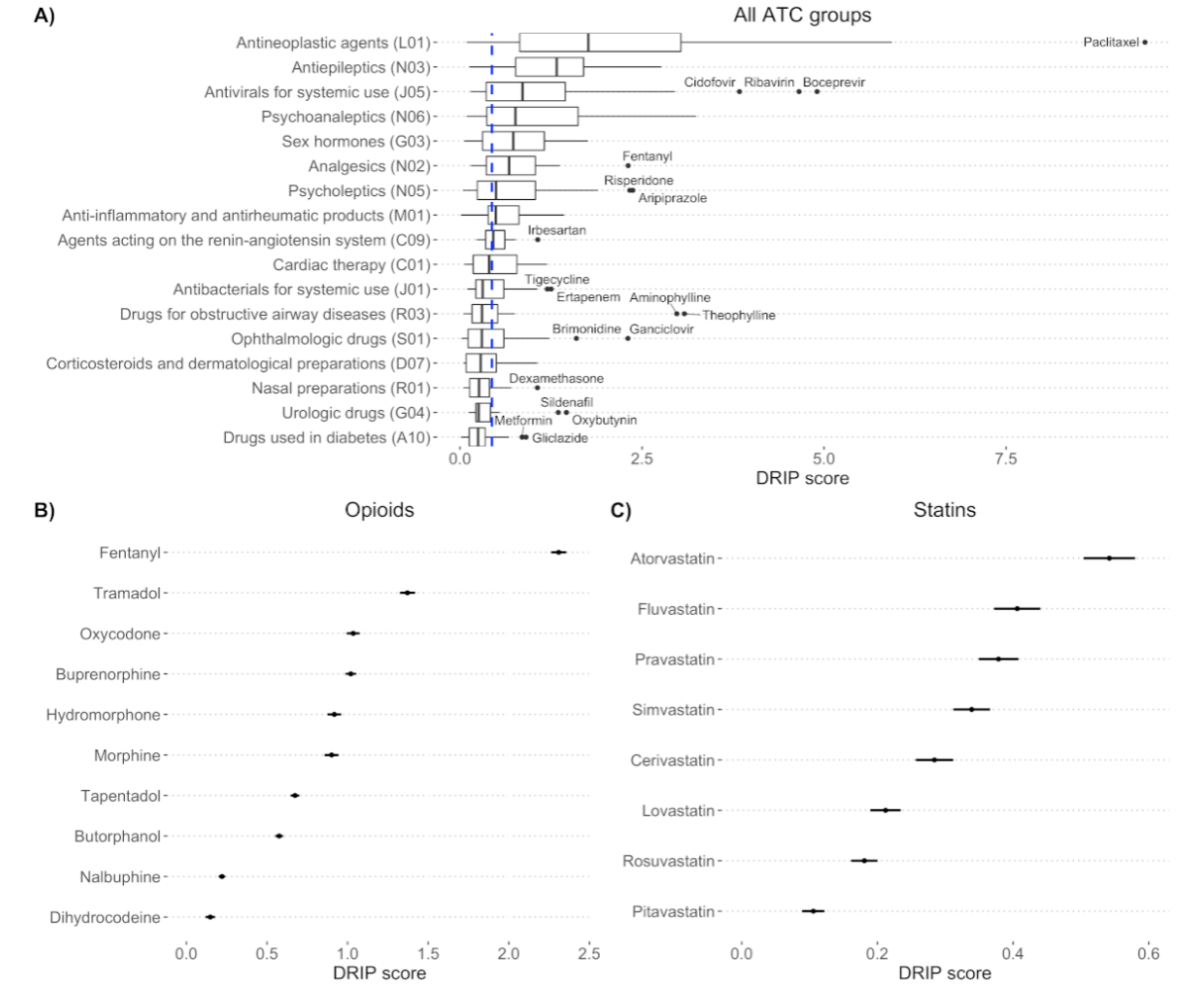
DRIP scores calculated using side effect severity and frequency. (A) DRIP scores (x-axis) for ATC classification system groups (y-axis) ordered by median severity with outliers labeled. The dashed blue line indicates the median DRIP score of 0.439 across all drugs. (B) DRIP scores (x-axis) for opioid class drugs (y-axis), as denoted by the N02A ATC group. Fentanyl, an extremely potent synthetic opioid, is ranked first as having the most severe side effect. (C) DRIP scores (x-axis) for statins (y-axis), as denoted by the C10AA ATC group. Atorvastatin, the strongest statin in broad use, is ranked to have the most severe side effect, whereas lower-efficacy statins are ranked as having less-severe side effects. ATC: Anatomical Therapeutic Chemical; DRIP: drug-risk profile.

We analyzed DRIP score distributions by the Anatomical Therapeutic Chemical (ATC) classification system for the subset of drugs with an ATC designation (Figure 5). Antineoplastic and antiepileptic drugs were the drug classes with the highest DRIP scores, indicating that the drugs in these classes have severe ADR profiles. Drugs used in the management and treatment of diabetes and urologic issues had the lowest DRIP scores, indicating that these drugs are relatively safe and have less-severe side effects. We examined individual drugs within the opioid class and found that fentanyl had a markedly higher DRIP score than other drugs in the same class (Figure 5). Drugs within the statin class were primarily below the overall median DRIP score (0.439), with only atorvastatin being markedly above the median DRIP score (Figure 5).

## Discussion

### Principal Findings

In this study, we present the SAEDR scores and quantitative estimates of the severity of 28,113 MedDRA ADR terms. The SAEDR scores have strong correlations with both crowdworker-based severity estimates and real-world ADR case outcome statistics. We generated these estimates using a network-based label propagation approach that required only a small percentage of the terms to be labeled. This study demonstrated the feasibility of using distantly supervised techniques such as label propagation to generate quantitative values for medical concepts.

Our approach enabled us to increase the number of ADRs with quantitative severity estimates from 2929 to 28,113, an almost tenfold increase, while minimizing the human time involved in generating the estimates. Our severity estimates can be updated routinely based on the recomputation of the embeddings and reseeding of the reference severe or benign ADRs. We noted that our severity estimates had a higher correlation with the aggregate crowdworker rankings (ρ=0.735) than the crowdworker rankings had among the three replicates in the original study (ρ=0.710). In addition, our estimates had a greater correlation with real-world outcomes such as FAERS cases resulting in death (ρ=0.595) than the crowdworker rankings (ρ=0.53). Thus, our severity estimates track with both human judgments of ADR severity and real-world outcomes of consequence to patient health.

We found that our SAEDR scores had a strong negative correlation, *ρ=*−0.748, with no case outcome being reported in the FAERS. We assigned a *No Outcome* label to the FAERS cases for which none of the available but relatively severe case outcomes had been reported. We took this as an indication that the FAERS cases lacking outcomes may be a result of cases having an outcome below the level of seriousness that is reportable in the FAERS. FAERS case outcome completeness could potentially be improved by creating a lower-severity category for case outcome documentation. We also noted that the SAEDR scores have a stronger correlation with serious outcomes in FAERS cases than with death outcomes. This can be partially explained by the fact that many more ADRs result in serious outcomes than in death, leading to poor differentiation of severity based on death for many ADRs where death is a rare or unobserved outcome.

In an effort to compare the impacts of using different methods to select the initial severe and benign ADR seed terms for label propagation, we compared crowdworker and real-world outcomes–based seed selections. We found that both methods resulted in consistently high severity scores for ADRs in the highest-severity SOC groups such as *Neoplasms* and *Cardiac Disorders*. We observed the largest differences in the *Product Issues* SOC group. Real-world outcomes–based seeds increased the severity of psychiatric disorders and social circumstances and decreased the severity of product issues. The importance and impact of psychiatric disorders may not be apparent to those not in the medical profession. Thus, it is possible that the crowdworkers did not perceive mental illness to be as severe as physical illnesses, leading to a bias in the rankings. The use of real-world outcomes–based rankings to select seeds led to more psychiatric disorders being included in the seed terms for the severity propagation. Similarly, the shifts in social circumstances and product issues are likely due to their limited presence or absence within the original crowdworker rankings because only seven ADRs in the social circumstances category were included in the 2929 ADRs ranked by crowdworkers, and no *Product Issues* ADRs were included in that ranking. This absence resulted in no ADRs from these groups being included in the initial seed set of labeled ADRs for the crowdworker-seeded method run, likely limiting the ability of the label propagation to accurately estimate the severity of these ADRs.

We found that the overall severity ordering of the SOC classes based on our SAEDR scores made intuitive sense. We were surprised by the ability of the label propagation approach to adjust the severity estimates based on particular key terms. For instance, the ADR terms for cancer (*Neoplasm*) ADRs are relatively similar on a lexical basis, but individual modifiers such as malignant and benign resulted in significantly different SAEDR scores for the respective groups of ADRs (Figure S3 of Multimedia Appendix 1). Similarly, examining groups of ADRs at different MedDRA hierarchy levels demonstrated relative severity estimates that were sensible to us, such as *Heart Failure Signs and Symptoms* having lower severity than the different types of actual heart failure.

We found a significantly higher estimate of severity among the ADRs that had been included in a boxed warning section than among those that had not. This offers further validation of the SAEDR scores because they are in agreement with past regulatory and drug labeling decisions. Our comparison of HLGTs with disproportionate rates between sexes revealed that male-associated ADRs were more severe. Notably, the SAEDR scores of the male-associated ADRs were not significantly different from those of the ADRs that have been included in a boxed warning. This is in agreement with previous work that highlighted the relatively higher severity of ADRs experienced by men [29,30].

We found that ADRs discovered in the postmarketing period are generally more severe than those discovered in the clinical trials based on our SAEDR scores. This indicates that postmarketing surveillance and ongoing regulatory discussions of risk-benefit trade-offs for particular drugs are necessary to keep the public safe [31]. These findings are not new because a recent study examining internal Food and Drug Administration data on drug labeling changes found that 35% of the ADRs added to drug labels were added to the boxed warnings and warnings and precautions sections [32]. Of the postmarketing ADRs, those involving more than one drug, as identified by TWOSIDES, were among those with the highest severity, indicating the increased risks associated with polypharmacy. This finding highlights the need for further research into the safety of polypharmacy because approximately half of the individuals prescribed a prescription drug are prescribed more than one concurrent medication [33].

One of the original aims of the pioneering study by Tallarida et al [7] was to create quantitative risk scores for individual drugs. Our DRIP scores are an attempt to do so, and we combined ADR severity with frequency information to generate a numerical estimate of a drug’s risk profile. We were able to generate DRIP scores for 968 drugs using ADR frequency data from the SIDER. Our DRIP scores have a modest Spearman correlation, *ρ=*0.377, with FAERS cases that resulted in death, where that drug was the primary suspect. Generally, correlations greater than 0.8 are considered strong, but this generally assumes that the comparison is with a gold standard ground truth. Because of a lack of an objective gold standard of DRIPs, we compared with FAERS case outcomes for the drugs in question, although the case outcomes are imperfect because of reporting bias and other issues. In addition, the DRIP scores were created using estimates of ADR frequencies from the SIDER that are primarily derived from clinical trial data. These ADR frequency estimates are affected by the dosage and underlying health of the clinical trial population, both of which are only sometimes reported. High-quality frequency estimates for postmarketing ADRs are essentially nonexistent, and many challenges exist to accurately estimating their frequency [29]. These issues limit the ability of our DRIP scores to accurately quantify risk. However, despite the limitations of the benchmark data and the ADR frequency information, the DRIP scores still demonstrate a modest correlation with real-world outcomes. We interpret this as an indication that our DRIP scores track with real-world outcomes and capture signals related to a drug’s safety profile that could be useful for downstream application. Further evidence of their validity is provided by the qualitative evaluation of the DRIP score distributions for the ATC groups. The relative distributions of the ATC groups make intuitive sense, with drugs used to treat cancer—antineoplastic drugs—having the most severe side effect profiles, whereas drugs used to treat diseases that are known to have safe and effective treatment options, such as diabetes, are ranked among the safest drug categories.

We present ADR severity estimates that track with both real-world outcomes and human perception, but these estimates are still limited. We used word embeddings trained on Reddit data, but other social media data could improve the severity prediction model. We used RedMed because the model was publicly available and contained many ADR terms of interest to this study. However, Reddit as a social media platform is skewed toward men and young people, who may discuss ADRs in different ways from the general population. Learning new embeddings from social media corpora generated by the patient groups most at risk for a given set of ADR experiences or from biomedical literature might improve model performance and address issues with demographic model biases.

FAERS case outcome proportions as a benchmark for severity are limited because of the outcomes being reported at the case level by the FAERS. Because of this case-level aggregation, the ADRs in the severe category may have inflated severity. For instance, symptoms of cardiac arrest may often be reported as separate ADRs for a FAERS case of cardiac arrest, resulting in symptoms that seem to be more severe than they are when occurring independently of the severe ADR with which they are associated.

Our results indicate that men reported more severe ADRs based on sex-specific ADRs identified in the study by Chandak et al [27]. Other research efforts have reported similar findings based on ADR-reporting databases [29], but there are potential sources of bias that could have affected this result. The data underlying the sex-specific ADRs identified in the study by Chandak et al [27] were derived from the FAERS. The study method focused on the analysis of a sex-balanced cohort of case reports, with cohort creation through propensity score matching of individuals. Propensity score matching can correct for factors included in the model, but it is unable to correct for underlying biases in reporting. It has been documented that there are higher rates of ADR reporting for female patients than for male patients, whereas male patients tend to have more severe outcomes reported [30]. It is likely that male patients are enriched for severe ADRs because of this reporting bias, and ADR reports should not be conflated with all ADRs experienced by individuals because of the low rate of ADR reporting [34]. Another limitation is that sex-specific ADRs were only reported at the HLGT level, resulting in SAEDR scores that were an average of all PTs within the HLGT. Figure 3 highlights the large range in terms of severity, even within a particular HLGT. It is possible that an analysis of sex-specific ADRs at the PT level would result in a different finding. Overall, although ADRs disproportionately reported in men are more severe than those disproportionately reported in women, more research is needed to reach a complete understanding of sex-specific differences in ADR severity.

Although we captured some nuances in ADR severity, such as benign versus malignant neoplasms, we only created SAEDR scores for the ADR terms contained in the MedDRA. However, while exploring the RedMed word embeddings, we observed that individual ADR terms were often contained in phrases indicating modified severity. For instance, a report of stomach pain might be modified with an adjective such as excruciating or mild. Although the MedDRA does not contain an intensity scale component for its terms, capturing this information from patients might further enable risk-benefit trade-off calculations. The approaches for general word sentiment assignment, similar to the one used in the study by Hamilton et al [22], could be repurposed here to assign severity to ADR modifier terms.

### Conclusions

In summary, we demonstrated that lexical networks and label propagation can be used quantitatively to estimate the severity of medical conditions. We showed the distributions of ADR severity among different groups of conditions, different groups of patients, and different ADR discovery time points. We combined our SAEDR scores with available ADR frequency data to generate quantitative DRIP scores and examined the distribution of the resulting DRIP scores. Our results (and future improved estimates) enable new quantitative analyses within the field of pharmacovigilance. To this end, we provide the complete set of SAEDR scores and DRIP scores in Multimedia Appendix 2 and Multimedia Appendix 3, respectively.

## Appendix

Multimedia Appendix 1 Supplementary figures and associated captions.

Multimedia Appendix 2 Severity of Adverse Events Derived from Reddit scores for 12,198 adverse drug reactions.

Multimedia Appendix 3 Drug-risk profile scores.

## Abbreviations

ADR: adverse drug reaction
ATC: Anatomical Therapeutic Chemical classification system
DRIP: drug-risk profile
FAERS: Food and Drug Administration Adverse Event Reporting System
HLGT: high-level group term
MedDRA: Medical Dictionary for Regulatory Activities
PT: preferred term
SAEDR: Severity of Adverse Events Derived from Reddit
SIDER: Side Effect Resource
SOC: System Organ Class
