# Supplementary material for: Quantifying the Severity of Adverse Drug Reactions Using Social Media: Network Analysis
Source: J Med Internet Res. 2021 Oct 21;23(10):e27714. doi: 10.2196/27714 (PMC8569532; doi:10.2196/27714)
Supplement: Multimedia Appendix 1 [file jmir_v23i10e27714_app1.pptx]

## Slide 1
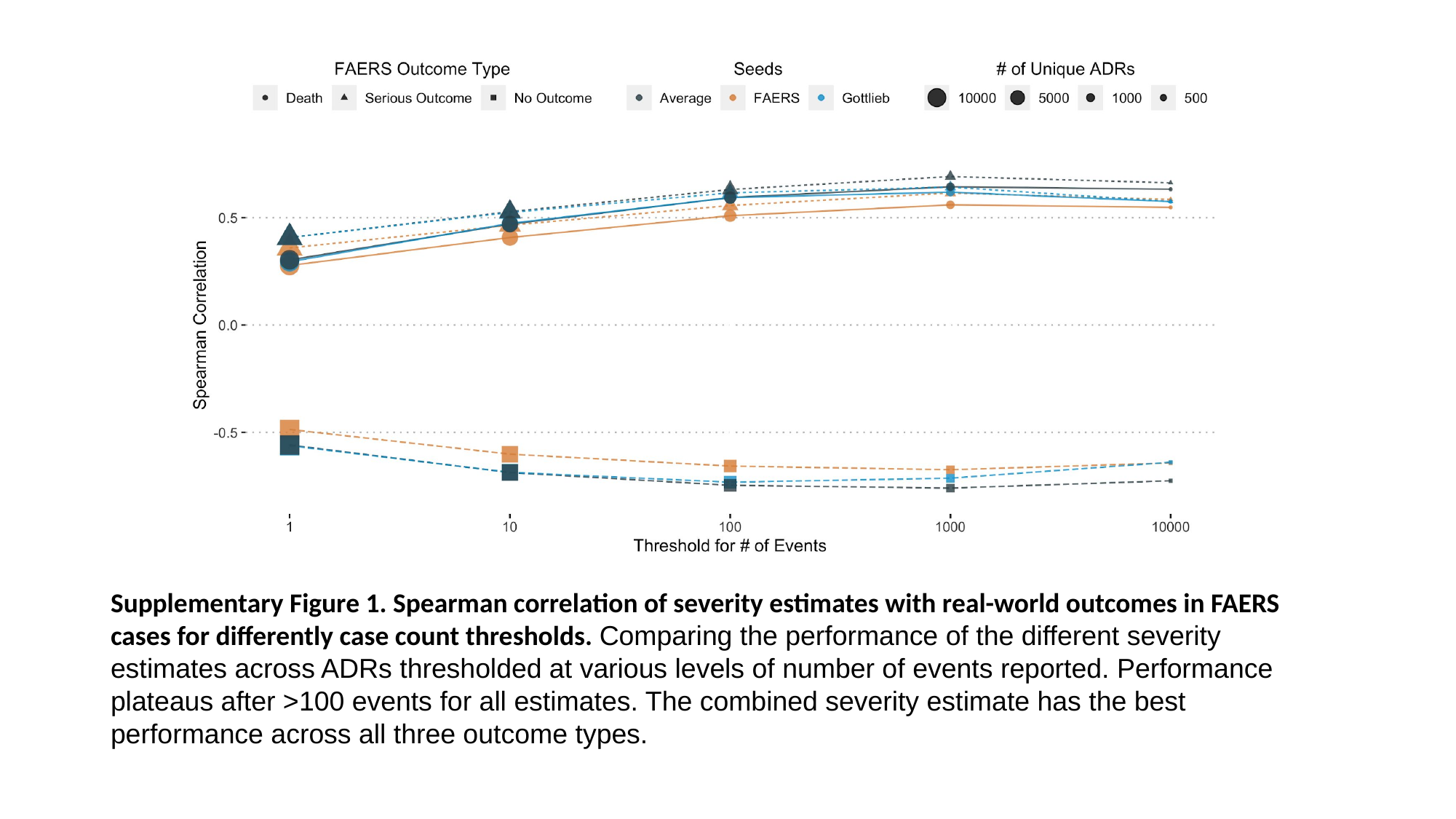

Supplementary Figure 1. Spearman correlation of severity estimates with real-world outcomes in FAERS cases for differently case count thresholds. Comparing the performance of the different severity estimates across ADRs thresholded at various levels of number of events reported. Performance plateaus after >100 events for all estimates. The combined severity estimate has the best performance across all three outcome types.

## Slide 2
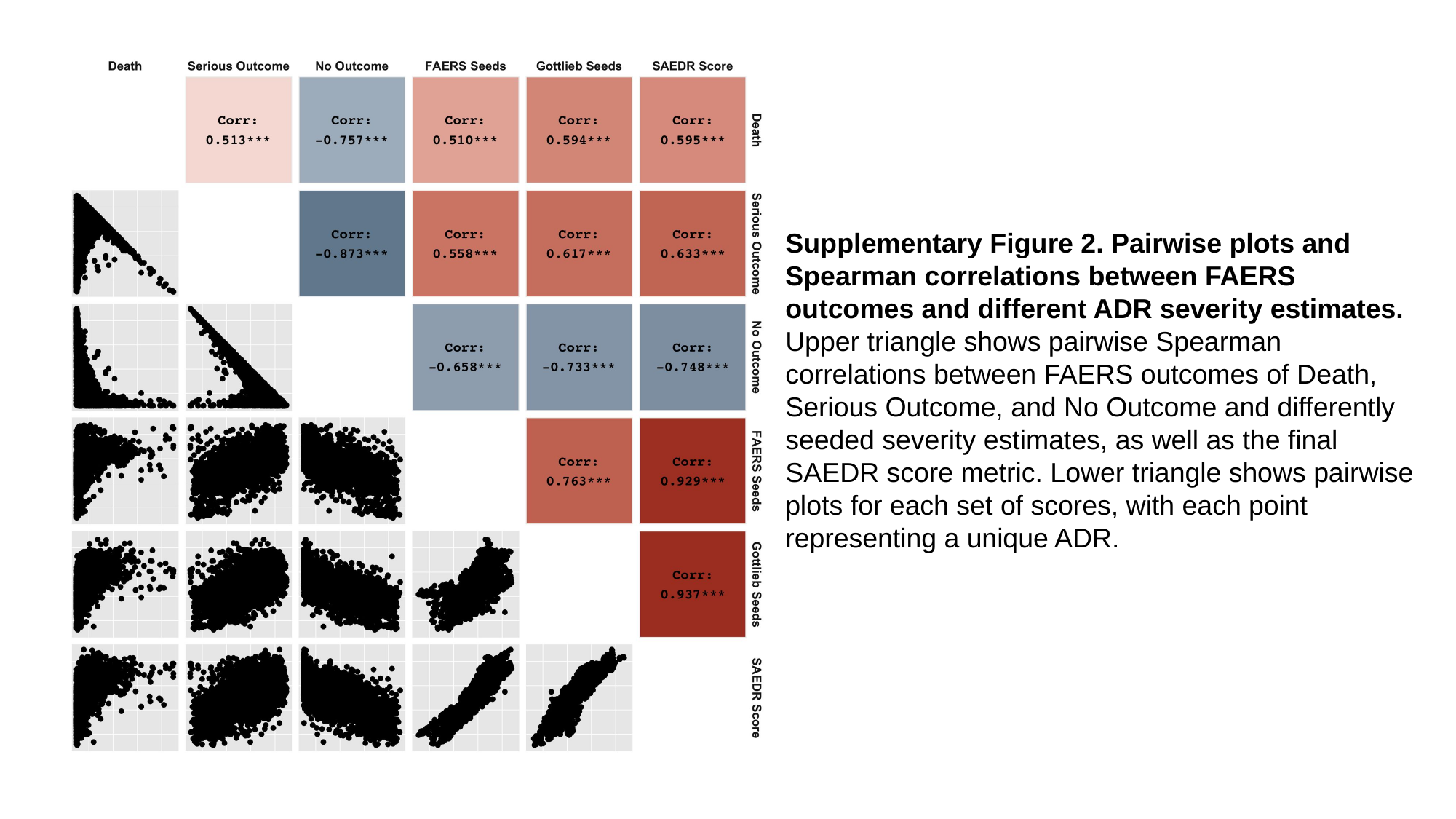

Supplementary Figure 2. Pairwise plots and Spearman correlations between FAERS outcomes and different ADR severity estimates. Upper triangle shows pairwise Spearman correlations between FAERS outcomes of Death, Serious Outcome, and No Outcome and differently seeded severity estimates, as well as the final SAEDR score metric. Lower triangle shows pairwise plots for each set of scores, with each point representing a unique ADR.

## Slide 3
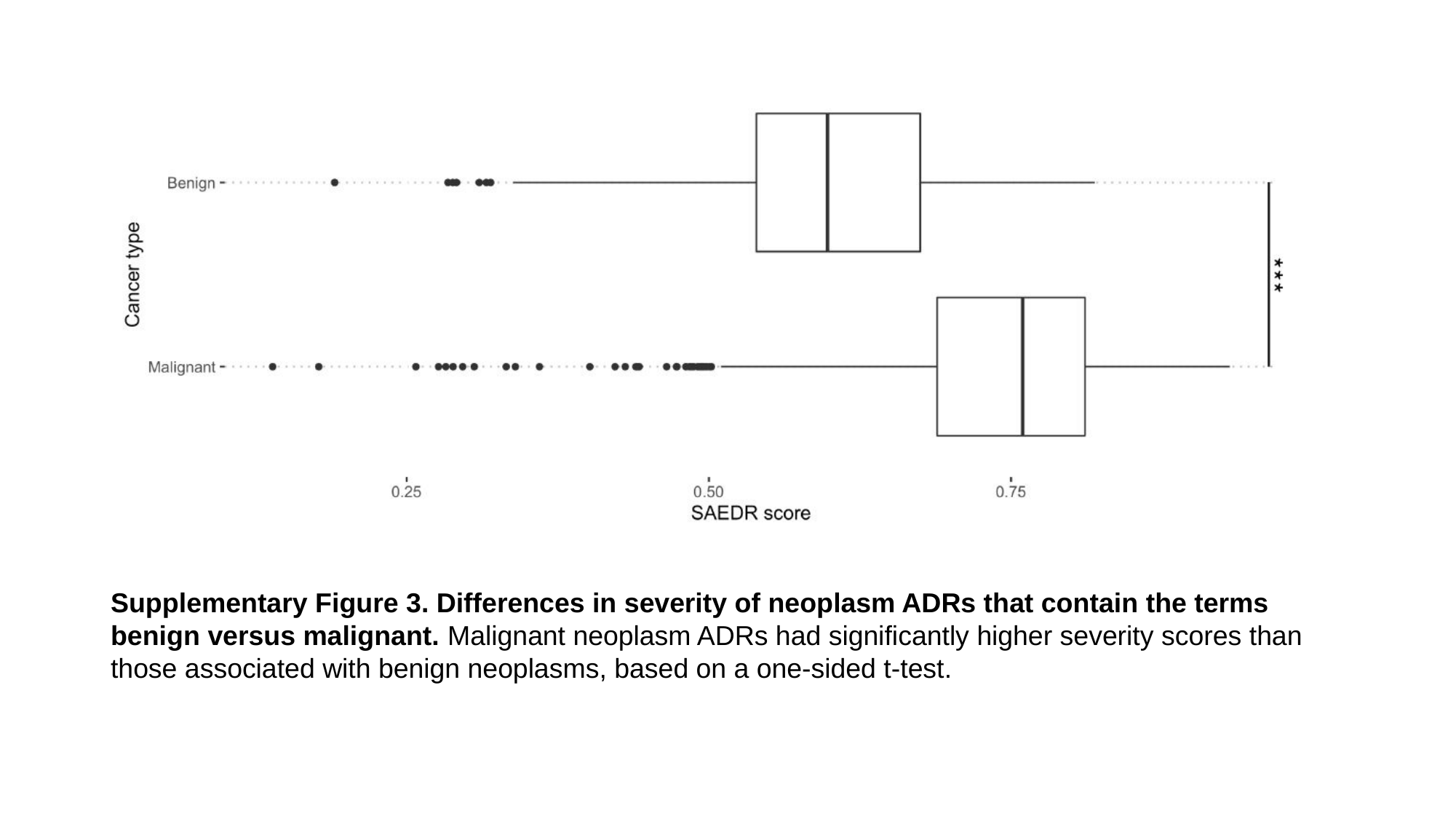

Supplementary Figure 3. Differences in severity of neoplasm ADRs that contain the terms benign versus malignant. Malignant neoplasm ADRs had significantly higher severity scores than those associated with benign neoplasms, based on a one-sided t-test.
